# Comparison of Markerless and Marker-Based Motion Capture Technologies through Simultaneous Data Collection during Gait: Proof of Concept

**DOI:** 10.1371/journal.pone.0087640

**Published:** 2014-03-04

**Authors:** Elena Ceseracciu, Zimi Sawacha, Claudio Cobelli

**Affiliations:** Department of Information Engineering, University of Padova, Padova, Italy; University of Zurich, Switzerland

## Abstract

During the last decade markerless motion capture techniques have gained an increasing interest in the biomechanics community. In the clinical field, however, the application of markerless techniques is still debated. This is mainly due to a limited number of papers dedicated to the comparison with the state of the art of marker based motion capture, in term of repeatability of the three dimensional joints' kinematics. In the present work the application of markerless technique to data acquired with a marker-based system was investigated. All videos and external data were recorded with the same motion capture system and included the possibility to use markerless and marker-based methods simultaneously. Three dimensional markerless joint kinematics was estimated and compared with the one determined with traditional marker based systems, through the evaluation of root mean square distance between joint rotations. In order to compare the performance of markerless and marker-based systems in terms of clinically relevant joint angles estimation, the same anatomical frames of reference were defined for both systems. Differences in calibration and synchronization of the cameras were excluded by applying the same wand calibration and lens distortion correction to both techniques. Best results were achieved for knee flexion-extension angle, with an average root mean square distance of 11.75 deg, corresponding to 18.35% of the range of motion. Sagittal plane kinematics was estimated better than on the other planes also for hip and ankle (root mean square distance of 17.62 deg e.g. 44.66%, and 7.17 deg e.g. 33.12%), meanwhile estimates for hip joint were the most incorrect. This technique enables users of markerless technology to compare differences with marker-based in order to define the degree of applicability of markerless technique.

## Introduction

Gait analysis is the systematic study of human walking, using the eye and the brain of experienced observers, augmented by instrumentation for measuring body movements, body mechanics, and the activity of the muscles [1]. In actual practice, gait analysis is used in orthopaedic hospitals and clinics to diagnose pathologies, design surgical operations, plan treatments for individuals with conditions affecting their ability to walk. In the last few years, a growing interest has been shown by the biomechanics community in novel markerless technologies, developed mainly in the fields of computer vision and for the entertainment industry [2]–[11]. The advantages that such methods could provide to the gait analysis field would be mainly the reduction in preparation time of the subjects and the absence of markers that could modify the naturalness of a subject's movement. As it is common to all new technologies, there is still the need for validation and standardization of the biomechanical models they comprise. Some efforts in this sense are made by the computer vision community, with the creation of public datasets that include marker data [12]. By admission of the authors however, recommendations for marker placement have not been strictly followed, as markers were attached to loose-fitting clothes [12]. Furthermore, the conventional marker set that has been employed provides less repeatable results than cluster based marker sets with 6-degrees-of-freedom biomechanical models. Finally, evaluation of algorithms' performance has been made from errors in joint centres (“virtual markers”) position, which do not represent the convention in clinical use. Description of functional joint angles, based on the precise anatomy of the subject and consistent with biomechanical societies' recommendations, has been so far neglected by markerless systems' developers; nonetheless, it is essential for the application of the latter in the clinical field.

The aim of this paper was to develop a method that enables users of markerless technology to compare differences with marker-based in order to define the degree of applicability of markerless technique in the clinical field. Therefore a procedure has been investigated for comparison of a state-of-the-art marker-based technique and a silhouette-based markerless approach, on lower limb joint angles estimation. Data has been acquired simultaneously with a commercial stereophotogrammetric system, saving to file the greyscale videos that are used for reconstruction of markers' 3dimensional (3D) trajectory.

Differences in calibration and synchronization of the cameras were excluded by applying the same wand calibration and lens distortion correction to both techniques. In order to calculate 3D joint angles with the markerless technique, technical frames of reference of relevant segments have been registered to anatomical ones. The marker-based technique adopted as a gold standard was chosen by considering that when using optoelectronic stereophotogrammetry, skin deformation and displacement causes marker movement with respect to the underlying bone. This source of errors in the estimation of joint kinematics is known as skin artifact [13]–[15]. Calibrated anatomical system technique (CAST) applied with the aid of singular value decomposition (SVD) algorithm as in [16]–[17] represents one of the techniques designed to minimize the contribution of this artifact and compensate for its effects [14]–[15].

## Methods

### 1.1 Ethic statement

The protocol was approved by the local Ethics Committee (of the University Polyclinic of Padova). Written informed consent was obtained from each participant.

### 1.2 Experimental set up

**Figure 1:**
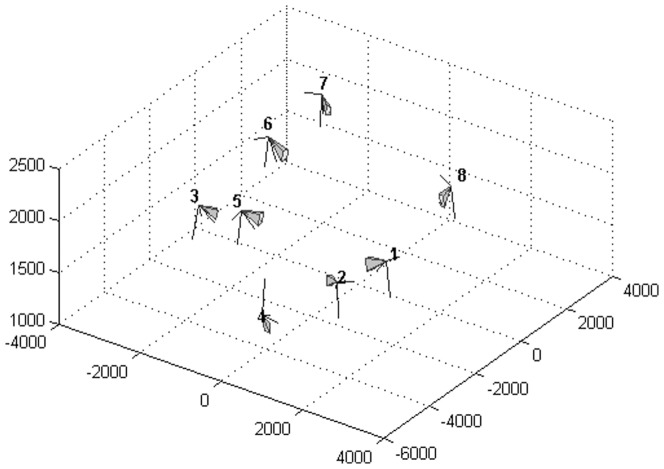
Cameras' position as resulting from extrinsic calibration. X, Y, Z axis are represented and each camera coordinates along axis are reported in [mm]. Each camera is identified by a number. Orange crosses correspond to additional infra-red illuminators that were not employed in visual hull reconstruction procedure.

An 8-camera SMART-D stereophotrogrammetric optoelectronic system (BTS S.r.l.) was employed to acquire experimental data. Acquisition rate was set to 200 Hz for marker data and 100 Hz for image data (one image frame every two was saved to file). Resolution of the CCD digital cameras was 640×480 pixels. Calibration was performed following manufacturer's recommendations: a rigid wand on which three markers are mounted is swept through the volume of interest, in a dynamic acquisition, for simultaneous calibration of intrinsic parameters, and relative position, of the video-cameras; a three-axes calibration grid is placed on the ground and acquired for determination of the global frame of reference. Position of the eight cameras is reported in Figure 1. Two additional infrared illuminators were placed close to the ground (positions indicated by the orange crosses in Figure 1), as to increase contrast between the subject and the floor. While placing the cameras, several requirements had to be taken into consideration. For markerless analysis, the cameras need to view the whole subject at all times, and from as most complementary views as possible [14]. On the other hand, for markers' reconstruction, each of them must be in view in at least two cameras, so multiple cameras should be placed to each side of the subject. The resulting configuration is a compromise between these different demands. Cameras 4 (frontal view) and 5 (sagittal view) are mainly dedicated to markerless analysis, while the others, though still useful for visual hull (VH) reconstruction, were placed according to recommendations for markers visibility. Only cameras 1–6 however were used for VH creation. A modified version of IORgait protocol [18] was used as in [19]–[20]: forty-eight 10-mm-diameter spherical markers have been used. A pointer, on which two markers are mounted at known distance, was used for anatomical calibration [19]–[20]. The manufacturer of the motion capture system provided a toolbox for Matlab for extraction from data files of calibration parameters and video data to be input into the markerless system. In order to test the applicability of the novel procedure for co-registration and comparison of markerless and marker-based gait analysis techniques, a healthy subject (female, age 26, BMI 20.9) was recruited. The model was generated either by means of a laser scan of the subject or through a static VH [10] as previously done by the authors in [20]. Both were adopted as input for model creation in the automatic model generation procedure as in [7]–[11].

The subject was wearing tight fitting clothes and a swim cap, and barefoot. After the anatomical calibration acquisitions, video capturing from the infrared cameras was activated. The subject was first asked to stand still in a reference (orthostatic) position, then to perform fifteen walking trials. Video acquisition of the sole background was also performed.

### 1.3 Markerless data processing

**Figure 2:**
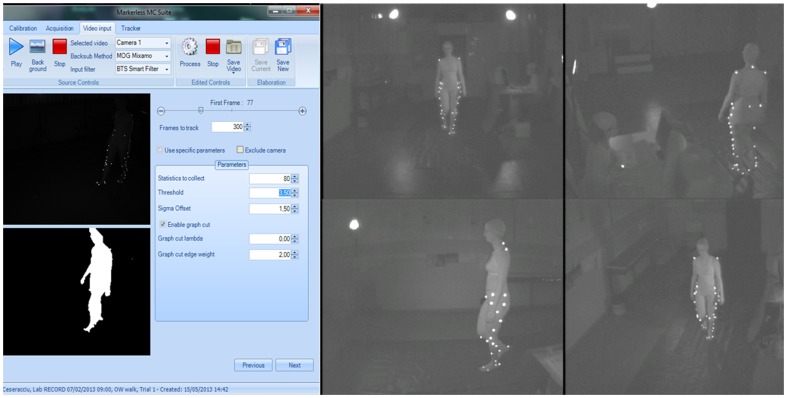
Example of background subtraction procedure: on the left side one frame from one of the acquired video sequences is reported: above subject with markers and below results of background subtraction. On the right side 4 views of the same grey-scale video sequence from 4 different cameras has been reported.

The background subtraction step is common among almost all markerless video based approaches [2]–[11]. Foreground/background segmentation is in general more difficult for grayscale images, since the chromatic component of an image has often more discriminative power than sole luminance. We try to take advantage however of the fact that this type of images represent, at each pixel, the intensity of light in the sole infrared band of the electromagnetic spectrum; we assume that the subject's skin and clothes will present greater response to infrared illumination than the surrounding background scene (see Figure 2).

**Figure 3:**
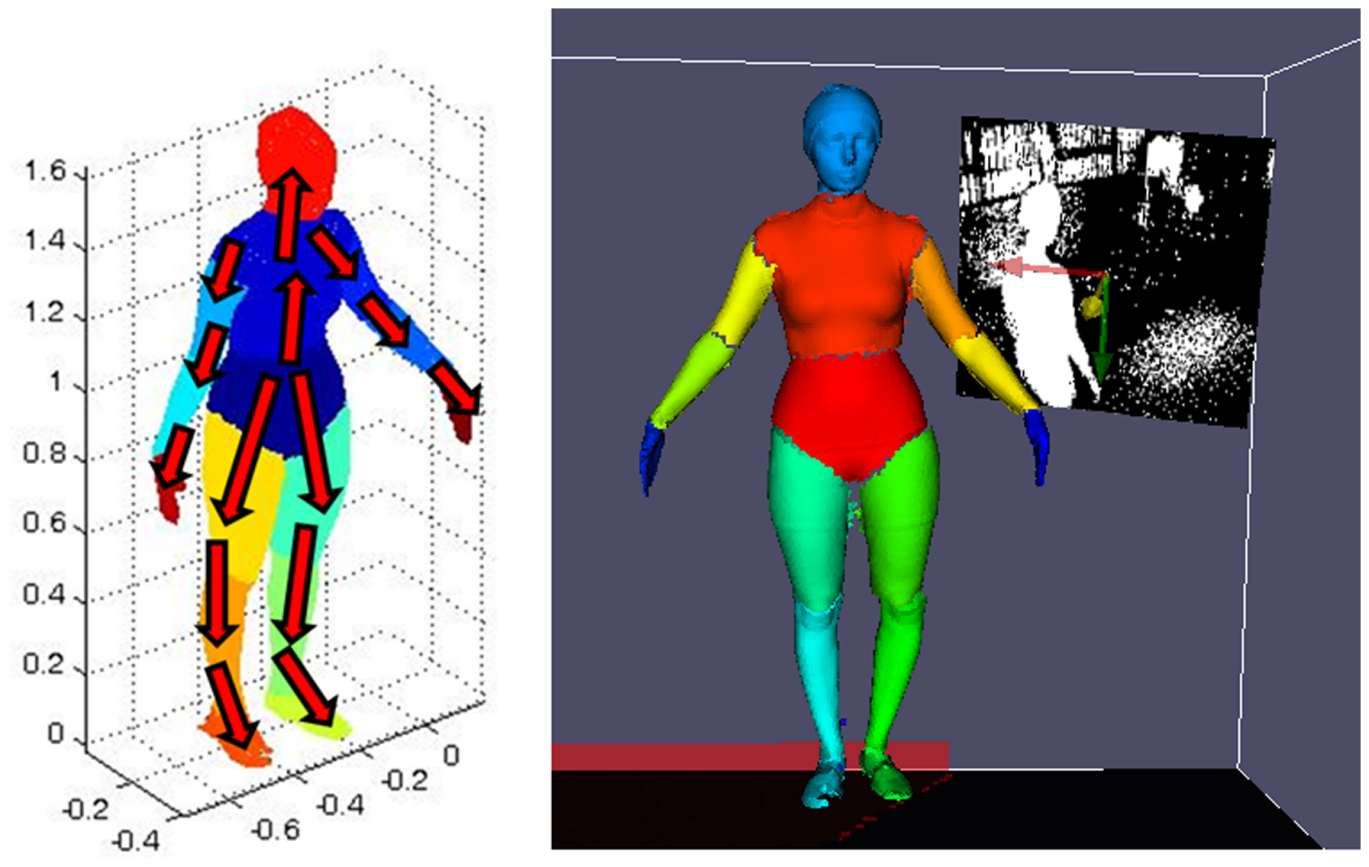
From left to right: tracking of the model as obtained from a static trial; scheme of the model kinematic tree (each arrow point from “parent” segment to “child” segment).

**Figure 4:**
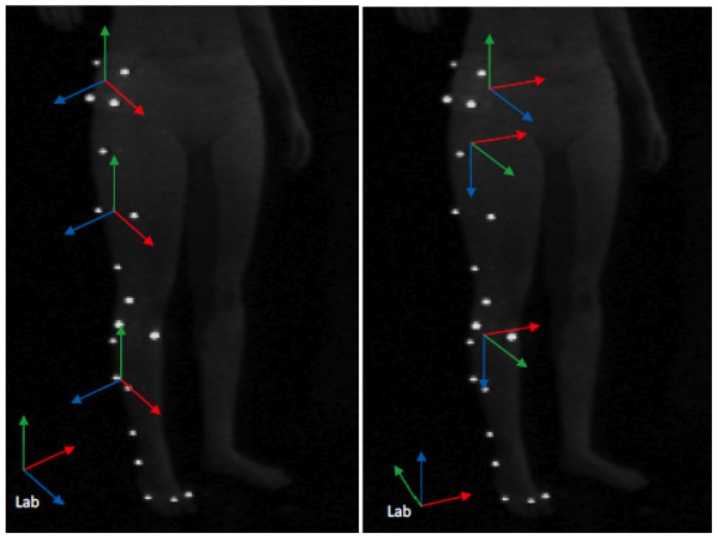
Global reference system, and technical reference systems for marker-based (left) and markerless techniques (right).

**Figure 5:**
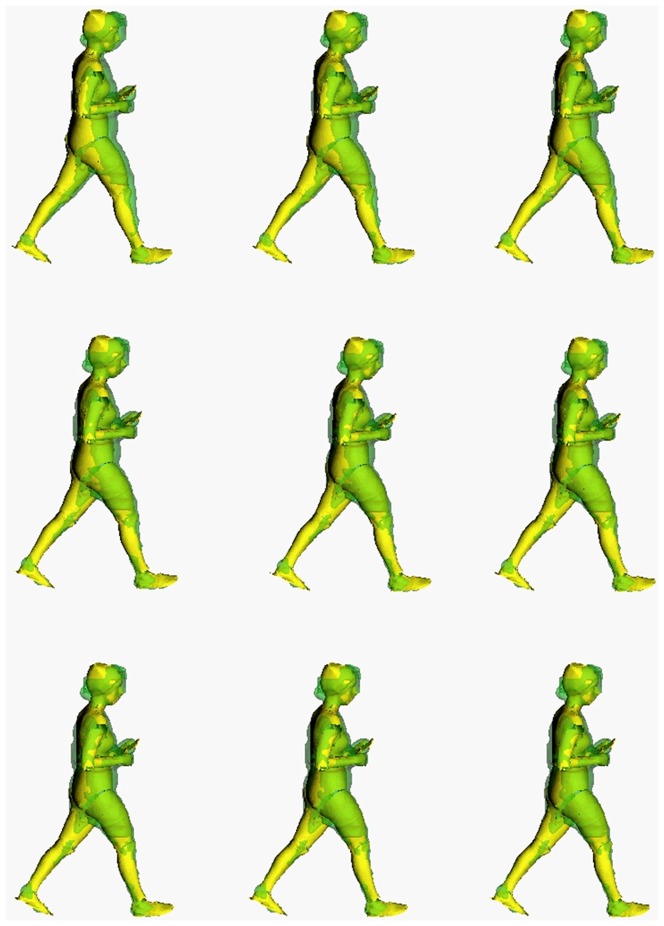
Example of nine iterations of the articulated-ICP algorithm for matching of the model (yellow) to visual hull data (green).

VH are created from silhouettes as indicated in [7], [9]–[10]. A slight modification was applied to the adopted model definition with respect to [7], [9]–[10] because the pelvis is chosen as root of the kinematic tree. The kinematic relationship between the segments is clarified by Figure 3. A slight modification has been introduced in the definition of segments' frames of reference, so that they could resemble more closely the anatomically-based ones employed for marker-based analyses as follows (see Figure 4): the longitudinal (Y) axis for thigh and shank segments is the axis connecting their parent and child joint centres; anterior-posterior (X) axis is the component of global anterior-posterior axis that is orthogonal to Y axis, and Z axis is perpendicular to the others; for pelvis segment, axes are parallel to global ones; foot segments' axes instead have been based on a principal component analysis (PCA) of the relative vertices on the mesh. The sequence of VHs is tracked employing the articulated-ICP algorithm described in [9] with a data-to-model approach (visual example shown in Figure 5); the roto-translation matrix defining the position and orientation in space of each body segment's embedded frame of reference is obtained.

### 1.4 Marker-based data processing

The protocol that has been employed as gold standard for segmental kinematics' measurement is a modified version of the IORgait protocol [18], and has been actively used in clinical studies [19]–[20]. It is based on Cappozzo et al. 1995 [16] that is a well recognized standard procedure for marker-based gait analysis. Anatomical calibration of some anatomical landmarks, either using a pointer or directly a marker, with respect to technical frames is involved [19]. This allows obtaining the global position of relative anatomical landmarks (ALs) from the pose estimated with marker-based as described by [19]. The pose of each cluster's embedded frame of reference is then estimated at each frame through an optimal least-square procedure based on SVD decomposition of cross dispersion matrix [17] which is considered one of the most robust methods in gait analysis [13]–[17]. Anatomical frames of reference defined in the IORgait protocol are based on the ones proposed by [16] and mostly consistent with relevant international recommendations [21]–[23]. From the pose of anatomical frames of reference, joint angles are calculated to describe the relative orientation of two adjacent body segments as in [17]–[23].

### 1.5 Comparison between markerless and marker-based

In order to compare the performance of markerless and marker-based systems in terms of clinically relevant joint angles estimation, the same anatomical frames of reference must be defined for both systems. This is a crucial aspect when considering that marker-based 3D joint angles estimation strictly depends from joint embedded frame of references definition [21]–[22], while markerless ones are only related to technical frames that are far from been easily interpreted in a clinical context or from enabling comparison with state of art 3D clinical gait analysis [16]–[22].

The procedure that has been followed exploits the anatomical calibration performed in the marker protocol in order to substitute the technical frame of markerless technique with anatomical one [16]–[17]. This procedure requires the tracking with both systems of a static acquisition of the subject. Pose estimation of segments' embedded frames of reference is then obtained and compared. For a segment *s* the relationship between its pose estimated with marker-based and markerless techniques can be represented by a 4×4 transformation matrix τ as in the following equations (eq. 1 and eq.2):
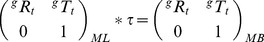
(1)

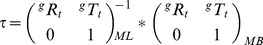
(2)where MB represents the marker based system, g represents the global reference system, ML represents the markerless system, Rt is the orientation matrix and Tt is the position vector estimated as in [16]–[17].

Assuming that this relationship is only representative of the difference in technical frame definitions, we exploit it in dynamic acquisitions to obtain an estimate of the marker-based technical frame from the markerless estimation (eq.3):
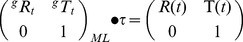
(3)


This allows obtaining the global position of relative ALs from the pose estimated with markerless in any instant of time (eq.4):
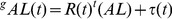
(4)


Segmental kinematics based on the ALs' trajectories thus reconstructed can be compared with those obtained through marker-based tracking of the dynamic acquisition.

Since no cluster of markers has been placed on the feet, calibration of anatomical landmarks has been performed on the anatomical frame of reference in the static acquisition.

A gait cycle for the right leg was analysed from each recorded trial. The vertical coordinate of a marker positioned on the heel was used to detect heel strike and toe off with the marker-based technique. Visual inspection of each frame of markerless tracking was used in order to detect heel strike and toe off events. Marker-based gait events detection was considered as a gold standard. The same events were adopted for processing data with both techniques. Joint angles calculated with marker-based and markerless technique (after applying equations 1–4) were compared, and the difference was evaluated in terms of root mean squared distance (RMSD). RMSD was evaluated for each time point of each gait cycle and then the mean RMSD over the 15 gait cycles was estimated. For the knee joint, only flexion extension angle was determined as it was proven to be the only one reliable when reconstructed by means of marker-based technology [13]–[15].

## Results

Hip, knee and ankle joint angles were calculated with both marker-based and markerless techniques and RMSD between measurements was obtained. In Table 1 mean and standard deviation (SD) of each joint angles were reported together with, minimum, maximum, mean and SD RMSD values calculated over all trials. Furthermore, mean RMSD values have been normalized to the corresponding range of motion calculated on a marker-based trial: the result is shown in the bottom row of the table. Best results are achieved for knee flexion-extension angle, with an average RMSD error, 11.75 deg, corresponding to 18.35% of the range of motion. Motion on the sagittal plane is estimated better than on the other planes also for hip and ankle, with respectively 44.66% (17.62 deg) and 33.12% (7.17 deg) errors. Estimates for hip joint are the most incorrect; error on the transverse plane reaches 129.57% of the relative marker-based range of motion. No significant differences were observed when generating the model by means of using the laser scan with respect to a static VH of the subjects in the comparison between markerless and marker-based joint angles; as already demonstrated by Corazza et al 2010.

**Table 1:** Description of root mean square distance (RMSD) values between angles calculated with the two techniques (markerless (mkl) and marker-based (mb)); mean, standard deviation (SD), minimum (Min) and maximum (Max) RMSD values are reported.

	Hip	Hip	Hip	Knee	Ankle	Ankle	Ankle
	AA	InEx	FE	FE	InEv	InEx	DP
Mean mkl (deg)	3.4	4.6	37.4	−21.6	1.6	−6.1	93.6
SD mkl (deg)	3.7	9.6	11.3	4.0	3.8	8.6	3.8
Mean mb (deg)	5.6	3.2	16.8	−20.9	−0.4	−10.6	42.6
SD mb (deg)	6.3	11.6	7.4	32.2	10.2	37.3	22.3
Mean RMSD (deg)	14.1	21.6	17.6	11.8	7.0	12.9	7.2
SD RMSD (deg)	2.3	9.3	8.5	2.5	3.6	7.0	1.8
Min RMSD (deg)	9.6	7.7	6.4	8.1	3.4	5.3	5.0
Max RMSD (deg)	17.3	34.7	29.0	16.0	13.8	29.4	11.0
RMSD % range of motion	91.5	129.6	44.7	18.3	54.8	88.2	33.1

Each joint rotation RMSD % of the marker-based range of motion is reported. Each joint rotations mean, standard deviation (SD), minimum (Min) and maximum (Max) values are also reported: Abduction- Adduction (AA), Flexion-Extension (FE), Internal- External Rotation (InEx), Inversion-Eversion (InEv), Dorsi-Plantarflexion (DP).

## Discussion

This project utilized a single subject design. The goal was to propose a methodology that enables assessment of the degree of applicability of markerless technique in the clinical field with respect to 3D joint motion estimation. In this context two crucial aspects had to be considered: first of all to compute 3D joints angles based on the same joints embedded reference systems with both techniques; second to enable definition of joint embedded reference systems, in agreement with international recommendation for gait analysis [22]–[23] through the markerless technique. Therefore lower limb 3D joints angles were estimated with markerless technique and compared with a state-of-the-art marker-based technique. It should be considered that a precise determination of anatomical embedded frames orientation is crucial for assessing joint kinematics reliability and interpretability [13]–[18]. In the context of marker placement a modified version of the IORgait protocol was adopted which is a common protocol established in routine clinical practice [18]–[20] and this version has been conceived as an attempt to reduce errors in the joint embedded frames definition and therefore in joint kinematics estimation [13]–[18], [25]. ALs position is estimated by applying the anatomical calibration technique, and the pose of each cluster's embedded frame of reference is estimated at each frame through an optimal least-square procedure based on SVD decomposition of cross dispersion matrix [17] (which is considered one of the most robust methods in gait analysis [13]–[17]). As a result, trajectories of ALs of feet, and of cluster points on pelvis and legs, are obtained. Anatomical frames of reference definitions and joint angles computations adopted herein are consistent with relevant international recommendations [21]–[22], differently than what previously performed in [12]. This was possible by applying equations 1–3 to the data simultaneously acquired during a static trial with both systems. To the authors' knowledge this represents an important step forward in state of art of markerless motion analysis. Hence comparison of results of the present paper with previous work is not straightforward. Only a previous contribution can be found that defined a method for identification of hip joint centers according to international standard for gait analysis [22] by using markerless motion capture [24].

It should be mentioned that skin artifacts are likely to play the main role in determining the accuracy of joints embedded frame, however in order to address this important aspect, a invasive-gold standard should have been provided (e.g. fluoroscopic acquisition). Therefore skin artifact contribution to joint embedded frames definition should be addressed by future work.

For the first time 3D joint angles of the lower limb were determined simultaneously with marker-based and markerless approaches by means of the same stereophotogrammetric system, that provided both calibration and data acquisitions. This is important, if we consider that source of errors in the comparison due to differences in calibration and synchronization of the cameras, can be excluded.

Other works that reported similar experiments, either employed two different systems for performing the two analysis [24], or did not use a stereophotogrammetric system for markerless motion capture [24]–[25]. Further differences should be pointed out: in one case the two systems were not synchronized and an ad hoc procedure was implemented in order to compare the data [24]; two different calibration procedures were used [24]–[25]; only 2D foot and ankle kinematics was provided [24]; in the other case only the kinematics of the hip joint centre was determined [25].

The actual results obtained by mean of this markerless technology from a gait analysis experiment seem to indicate that level of accuracy and robustness is still not sufficient in comparison with marker-based one. Estimates for hip joint are far from been acceptable in a clinical context together with the ones relatives to the motion on the transverse plane, thus weakening the possibility of evaluating all the 3 rotational degree of freedom required to describe each joint 3D motion. However it could be argue that when considering effect of tissue artifact on marker-based motion analysis, a more robust gold standard could provide different results (e.g. fluoroscopic gold standard). Therefore future developments should considered comparison with different gold standard thus excluding the contribution of skin artifact in joints angles estimation [13]–[15]. With this respect, some problems were identified within the present experimental set up which might have affected markerless analysis results: excessive “phantom volume” artifacts [2]–[11] at the level of the pelvis that may lead to errors in the estimates for hip joint angles; artifacts in the background subtraction caused by self-shadowing of that area in both midstance and midswing phases; rigid-body matching of the model surface of the foot to the foot in the VH (caused mainly by midfoot-forefoot flexion) can easily yield to an estimate of ankle joint angles which may differ from that based on markers. In this context the technique for hip joint centre definition proposed in [23] could minimize errors in hip joint computation. Additional factors, intrinsic to the comparative nature of this experiment, may have affected the results of markerless estimation. For instance, camera placement was conditioned by the requirements imposed by markers' visibility. The presence of the markers attached to the skin of the subject deformed the silhouettes and consequently the VHs. Nevertheless only six cameras were available for VH reconstruction, which results in reducing of two cameras the optimal number of cameras (8 cameras, according to [11]); however the present set up was able to fulfil the main requirement for VH creation which is that each camera views the whole subject at all times, and from as most complementary views as possible (see Figure 2) [11]. Accurate measurement of human body kinematics was obtained using a subject specific model generated through static VH or a laser scan. Results of the comparison between joints rotation estimated with both techniques were not influenced by the procedure adopted for model generation as already reported by Corazza [9], therefore the present methodology can be adopted avoiding requiring of expensive dedicated hardware like a laser scanner. Oppositely when considering the marker-based technique 6 cameras allowed only to determine one leg 3D kinematics, due to the constraint of cameras placement for markerless technique. Therefore the application of markers was limited to one leg. Nevertheless a larger sample subjects should be acquired in order to generalize results of the present experiment.

With respect to the background subtraction step it is common among almost all markerless video based approaches. For relatively less controlled situations, such as clinical gait laboratories where multiple instruments are used that remain in view of the cameras, a general approach is used: a reference background image, where no subjects are present, is taken and compared to each frame of the video sequence. Regardless of the actual technique employed for background/foreground segmentation, only the information relative to the shape of the subject is retained from the images. Each frame image is binarized assigning, for example, the value 0 (black) to all the background pixels and the value 1 (white) to all the foreground pixels. Then morphological operations are performed, such as dilation and erosion (*binary closure*) [2]–[5], in order to get rid of spurious pixels or holes in the foreground patch. We try to take advantage however of the fact that our images represented, at each pixel, the intensity of light in the sole infrared band of the electromagnetic spectrum. The main problems within this technology and this approach lie in the limited robustness to the presence of shadows cast by the subject on the floor and on themselves.

It could be questioned that only the data of one subject were analysed, however this finds agreement with the state of art of gait analysis kinematics variability assessment studies [24]–[26].

Care should be taken when generalizing the findings of this study. It is likely that differences will exist in the relative contributions of the sources of measurement error when a wider cohort of subjects will be acquired. Finally when taking into account markerless application in the clinical field, this technique is still debated. This is mainly due to a limited number of papers dedicated to the comparison with the state of the art of marker based motion capture, especially in term of repeatability and accuracy in the estimation of the 3D joint rotations. The present method can be used for further testing and developing of silhouette-based markerless techniques. Its main advantage is the possibility to use state-of-the-art marker-based data as gold standard, without any difference in the definition of anatomical reference frames. The ability to perform different types of analysis with the same commercial system could be of use to gait laboratories, which could choose between one system or the other (or an hybrid version) in order to apply to markerless technique international recommendations on joint angles estimation. However further experiments should be performed in order to optimize the camera set up by increasing the number of cameras, and by trying to avoid limitations of the present contribution in term of VH deformation.

## Conclusion

Description of functional joint angles by means of a markerless technique, based on the precise anatomy of the subject and consistent with biomechanical societies' recommendations, has been enabled. Results of the present paper showed that meanwhile joint angles rotations were found comparable on the sagittal plane, their estimation on the transverse plane was not sufficiently precise to allow application in the clinical field. However in evaluating the results reported herein, limits of the experimental setup should not be neglected, together with their possible impact on error estimation. The possibility to adopt markerless motion capture technique in the gait analysis field is highly south if we consider that it can provide the reduction in preparation time of the subjects and the absence of markers that could modify the naturalness of a subject's movement. However by considering that in actual practice, gait analysis is mainly used in orthopaedic hospitals and clinics, the method developed herein can be used for assessing markerless' joint kinematics reliability and interpretability by applying a precise determination of anatomical embedded frames orientation.
